# Deep‐Learning Generated Synthetic Material Decomposition Images Based on Single‐Energy CT to Differentiate Intracranial Hemorrhage and Contrast Staining Within 24 Hours After Endovascular Thrombectomy

**DOI:** 10.1111/cns.70235

**Published:** 2025-01-24

**Authors:** Tianyu Wang, Caiwen Jiang, Weili Ding, Qing Chen, Dinggang Shen, Zhongxiang Ding

**Affiliations:** ^1^ Department of Radiology, Affiliated Hangzhou First People's Hospital Westlake University School of Medicine Hangzhou China; ^2^ School of Biomedical Engineering ShanghaiTech University Shanghai China; ^3^ Zhejiang Chinese Medical University Hangzhou China; ^4^ Shanghai United Imaging Intelligence Co. Ltd. Shanghai China

**Keywords:** deep learning, dual‐energy CT, endovascular thrombectomy, generative adversarial networks, hemorrhagic transformation, material decomposition images, postinterventional cerebral hyperdensity, stroke

## Abstract

**Aims:**

To develop a transformer‐based generative adversarial network (trans‐GAN) that can generate synthetic material decomposition images from single‐energy CT (SECT) for real‐time detection of intracranial hemorrhage (ICH) after endovascular thrombectomy.

**Materials:**

We retrospectively collected data from two hospitals, consisting of 237 dual‐energy CT (DECT) scans, including matched iodine overlay maps, virtual noncontrast, and simulated SECT images. These scans were randomly divided into a training set (*n* = 190) and an internal validation set (*n* = 47) in a 4:1 ratio based on the proportion of ICH. Additionally, 26 SECT scans were included as an external validation set. We compared our trans‐GAN with state‐of‐the‐art generation methods using several physical metrics of the generated images and evaluated the diagnostic efficacy of the generated images for differentiating ICH from contrast staining.

**Results:**

In comparison with other generation methods, the images generated by trans‐GAN exhibited superior quantitative performance. Meanwhile, in terms of ICH detection, the use of generated images from both the internal and external validation sets resulted in a higher area under the receiver operating characteristic curve (0.88 vs. 0.68 and 0.69 vs. 0.54, respectively) and kappa values (0.83 vs. 0.56 and 0.51 vs. 0.31, respectively) compared with input SECT images.

**Conclusion:**

Our proposed trans‐GAN provides a new approach based on SECT for real‐time differentiation of ICH and contrast staining in hospitals without DECT conditions.

## Graphical abstract

This study developed an advanced trans‐GAN using deep learning to generate synthetic material decomposition images from input single‐energy CT images for real‐time discrimination and diagnosis of intracranial hemorrhage and contrast staining within 24 h after endovascular thrombectomy.

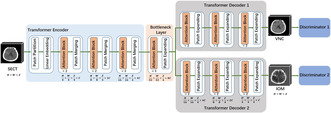

## 1 Introduction

Acute ischemic stroke (AIS) is a leading cause of disability and mortality worldwide [1]. The safety and efficacy of endovascular thrombectomy (EVT) have been confirmed in multiple randomized controlled trials, and EVT is now recommended as the first‐line treatment for AIS with large artery occlusion [2, 3, 4]. However, postinterventional cerebral hyperdensity (PCHD) is a common finding in noncontrast CT (NCCT) examinations after EVT due to blood–brain barrier injury. PCHD is usually composed of contrast staining (CS) alone under the circumstance of the limited injury confined to the endothelial cell layer, whereas blood extravasation, that is, hemorrhagics transformation (HT), can occur in the case of the degraded basal lamina [5, 6, 7]. As HT is one of the most serious post‐EVT complications in AIS patients, it is crucial to differentiate intracerebral hemorrhage (ICH) from contrast staining (CS) during the early post‐EVT period to guide follow‐up therapy and improve prognosis [8]. However, as both pathologies appear as high‐density shadows on single‐energy CT (SECT), it is difficult to distinguish between them in a single examination, and at least one re‐examination is required to obtain the diagnosis [9, 10]. Dual‐energy CT (DECT) addresses the issue of CS mimicking or concealing ICH on conventional CT images by using three‐material decomposition. It enables real‐time identification of ICH and CS by separating iodine and blood in material decomposition images (MDI), such as the iodine overlay map (IOM) and virtual noncontrast (VNC) images [11]. However, its application in clinical practice is still limited, primarily due to the higher cost of the scanner. Thus, searching for a new method that could generate synthetic MDI based on SECT images may be of great importance.

Recently, deep learning has been increasingly applied in medical imaging. Some studies have applied deep learning to the translation tasks between DECT and SECT. For example, scholars have previously attempted to generate matched CT images of another tube voltage based on CT images of a single tube voltage to simulate DECT original images and improve the quality of the material decomposition [12, 13]. Kawahara et al. [14] used GAN‐based CNN architectures to generate material decomposition images of bone (water) and fat (water) from 120 kVp SECT equivalent images. The success of these studies directed us toward deep‐learning techniques to generate synthetic MDI from SECT for PCHD diagnosis. To this end, we recently proposed a transformer‐based generative adversarial network (trans‐GAN), which adopts the vision transformer layers instead of traditional convolutional layers and thus can capture spatial dependencies in SECT images [15]. In this study, we evaluated the proposed method by comparing the efficacy of diagnosing PCHD by SECT with that of synthetic VNC (sVNC) and IOM (sIOM).

## 2 Methods

This study was approved by the local ethics committee. Due to the retrospective nature of the study, the requirement for informed consent was waived.

Between May 2020 and May 2022, 139 patients from the Affiliated Hangzhou First People's Hospital, Westlake University School of Medicine (Center A), and 25 patients from Yiwu Central Hospital (Center B) were consecutively included in the study. The inclusion criteria were as follows: (1) AIS diagnosed according to the 2019 AHA/ASA AIS guidelines; (2) patients with indications for EVT that received treatment; (3) those who underwent NCCT examination within 24 h after EVT (Center A used DECT, Center B used SECT). Exclusion criteria included: (1) post‐EVT NCCT showing no PCHD; (2) presence of artifacts affecting diagnosis; and (3) absence of images suitable for definitive diagnosis. Subsequently, complete NCCT image data containing PCHD within 24 h after EVT were collected for the enrolled patients. Additionally, for each patient, DECT data including matched IOM, VNC, and simulated conventional CT (sCCT, equivalent to 120‐kV conventional single‐energy CT) images were also collected. Finally, 237 cases of DECT data from 139 patients in Center A and 26 cases of SECT data from 25 patients in Center B were included. The difference in the number of scans performed within the first 24 h post‐EVT between the two centers is primarily due to variations in CT equipment and follow‐up strategies. The DECT data from Center A were randomly split into training and internal validation sets (4:1) using stratified random sampling to ensure the two sets had the same proportion of ICH. The SECT data from Center B were used as an independent external validation set. The diagnostic criteria for PCHD vary depending on the imaging modality (DECT/SECT). For DECT data, the classical definition of discriminating between CS and ICH based on the VNC and IOM images has been described in detail in a previous study [11, 16, 17, 18, 19]. However, due to the unknowable property of real‐time diagnosis of PCHD on SECT images, for the data from Center B, the results of NCCT re‐examination 2–3 days post‐EVT were used as the diagnostic criteria: the persistent presence or enlargement of PCHD on the follow‐up images indicates the presence of ICH, while if the PCHD is obviously absorbed or disappear, the diagnosis is CS [7, 20, 21]. The NCCT scan parameters for the two centers are listed in the Supporting Information.

### 2.1 Data Preprocessing

Horizontal flipping and rotation were applied for data augmentation during training. All images were resampled to a voxel spacing of 1 × 1 × 1 mm^3^ with the size of 256 × 256 × 128, and their intensity was normalized within [0, 256] by min‐max normalization. Moreover, to further augment the training samples and reduce the usage of GPU memory, the original image was randomly cropped to the size of 96 × 96 × 96 as input.

### 2.2 Network Architecture and Training

Our goal is to generate synthetic MDI, that is, sVNC and sIOM from SECT for diagnosis, with a focus on accurately generating high‐density regions. To achieve this, we enhance the generative adversarial network (GAN) by incorporating the visual transformer [22] technique, allowing the network to automatically prioritize important regions of the image. This results in the trans‐GAN, our proposed approach.

**FIGURE 1:**
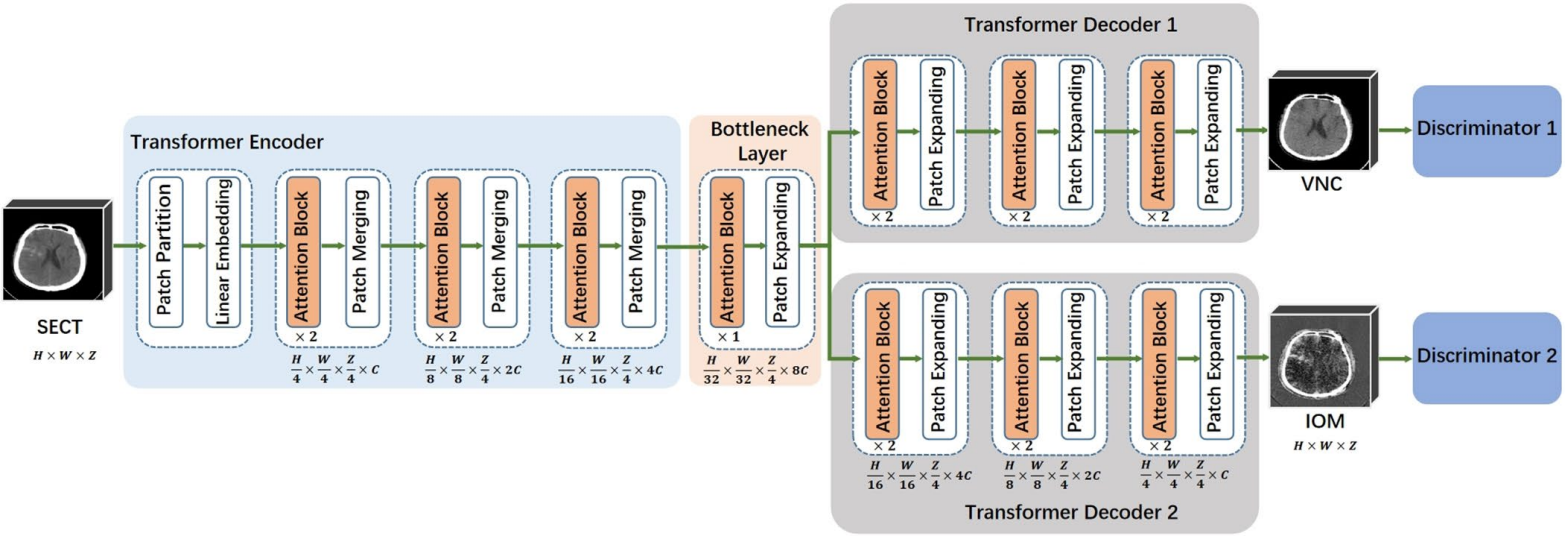
Illustration of proposed transformer‐based Generative Adversarial Network which contains (1) a transformer encoder to extract features from input image, (2) two decoders progressively upsample features to obtain the synthetic VNC and IOM predictions, and (3) two discriminators to provide adversarial losses. IOM, iodine overlay map; SECT, single‐energy CT; VNC, virtual noncontrast.

As shown in Figure 1, trans‐GAN consists of a transformer encoder, a bottleneck layer, two task‐specific transformer decoders, and two discriminators. When given a 3D SECT image, its features that contain both global and contextual information are first extracted by the encoder, and then pass through the bottleneck layer and enter into two decoders. The final predictions for sVNC and sIOM from the decoders are evaluated by the two discriminators to assess image quality. We provide the detailed architecture of each component in trans‐GAN below:

The transformer encoder is a pyramidal backbone [23] that contains four blocks, where the {1st} block consists of a patch partitioning layer along with a linear embedding layer while the {2nd, 3rd, 4th} blocks consist of two successive attention blocks followed by a patch merging layer. These blocks successively decrease the resolution and double the channel dimensionality of input, jointly producing a hierarchical representation. The bottleneck layer connects the encoder and decoders, consisting of a T1 block followed by a patch‐expanding layer. There are two transformer decoders with the same structure to predict VNC and IOM, respectively. The decoder contains three cascaded blocks, each consisting of two successive attention blocks and a patch‐expanding layer. These blocks increase the resolution and halve the channel dimension of the features, producing a hierarchical representation corresponding to the encoder. The output from the last block is passed to a linear layer to obtain the final H × W × Z × 1 prediction. Furthermore, two discriminators have the same architecture of four convolutional layers and a fully connected layer.

Denoting the predicted sVNC and sIOM as ${\hat{y}}_{\mathrm{VNC}}$ and ${\hat{y}}_{\mathrm{IOM}}$, our trans‐GAN can be trained with the loss functions as follows:

$$L_{\mathrm{VNC}}=L_{l1}\left({\hat{y}}_{\mathrm{VNC}},y_{\mathrm{VNC}}\right)+L_{d1}\left({\hat{y}}_{\mathrm{VNC}},y_{\mathrm{VNC}}\right),$$

$$L_{\mathrm{IOM}}=L_{l1}\left({\hat{y}}_{\mathrm{IOM}},y_{\mathrm{IOM}}\right)+L_{d2}\left({\hat{y}}_{\mathrm{IOM}},y_{\mathrm{IOM}}\right),$$

where $L_{l1}$ is the L1 loss, and $L_{d1}$ and $L_{d2}$ are the adversarial losses, while $y_{\mathrm{VNC}}$ and $y_{\mathrm{IOM}}$ are the ground truths. During the training, we employ the GradNorm [24] technique to dynamically adjust the training weights of $L_{\mathrm{VNC}}$ and $L_{\mathrm{IOM}}$ avoid overfitting.

All experiments were performed on the PyTorch platform using two NVIDIA Tesla A100 GPUs. During the training process, we used the Adam optimizer with *β*_1_ = 0.9, *β*_2_ = 0.999, and the initial learning rate was set to 10^−3^ whose decay is scheduled by Plateau Scheduler1 (factor = 0.5, patience = 5) based on the average training loss of the last 50 iterations. The batch size was set to 4 and the number of training iterations was set to 200.

To assess the superiority of trans‐GAN, we compared it with several state‐of‐the‐art generative networks, including autocontext CNN (AutoCNN) [22], cGAN [25], MedGAN [26], and AutoGAN [27]. The implementation details of each comparison method are provided in the Supporting Information. For a fair comparison, all networks were trained with the same data and settings.

### 2.3 Image Quality Assessment

To evaluate the synthetic MDI, we used two commonly employed metrics in generative tasks: peak signal‐to‐noise ratio (PSNR) and structural similarity (SSIM). The definitions of these metrics are provided in the Supporting Information.

### 2.4 Clinical Value Assessment: Image Interpretation

In addition to quantitative assessment, we also qualitatively assessed the sVNC and sIOM images. Two radiologists (with > 5 years of experience in neuroimaging diagnosis) independently reviewed the input images (sCCT/SECT) first, followed by the sVNC and sIOM images, to interpret the results of the validation set and the images generated by the network. During each interpretation, a differential diagnosis between ICH and CS was made for each PCHD, with any disagreements resolved by consensus. Another neuroradiologist (with 30 years of experience in acute stroke imaging) independently reviewed the images used to define the diagnosis of the internal and external validation groups. His interpretation results were then used as the gold standard for final diagnosis. All radiologists were blinded to the clinical information.

### 2.5 Statistical Analysis

SPSS package (Version 25.0, IBM Corp, Armonk, New York, USA) and MedCalc software (Version 16.8, Ostend, Belgium) were used for statistical analyses. Continuous values are presented as mean ± standard deviation or median with interquartile ranges, while categorical variables are presented as numbers and percentages. Sensitivity, specificity, positive predictive value, negative predictive value, accuracy, and receiver operating characteristic curve area under the curve (AUC) were calculated with 95% confidence intervals (CIs). The DeLong test was used to compare the AUC for differentiating ICH from CS using the input SECT images versus the sIOM and sVNC images generated by the network. Unweighted *κ*‐values with 95% CIs were calculated to assess the level of inter‐reader agreement.

## 3 Results

**FIGURE 2:**
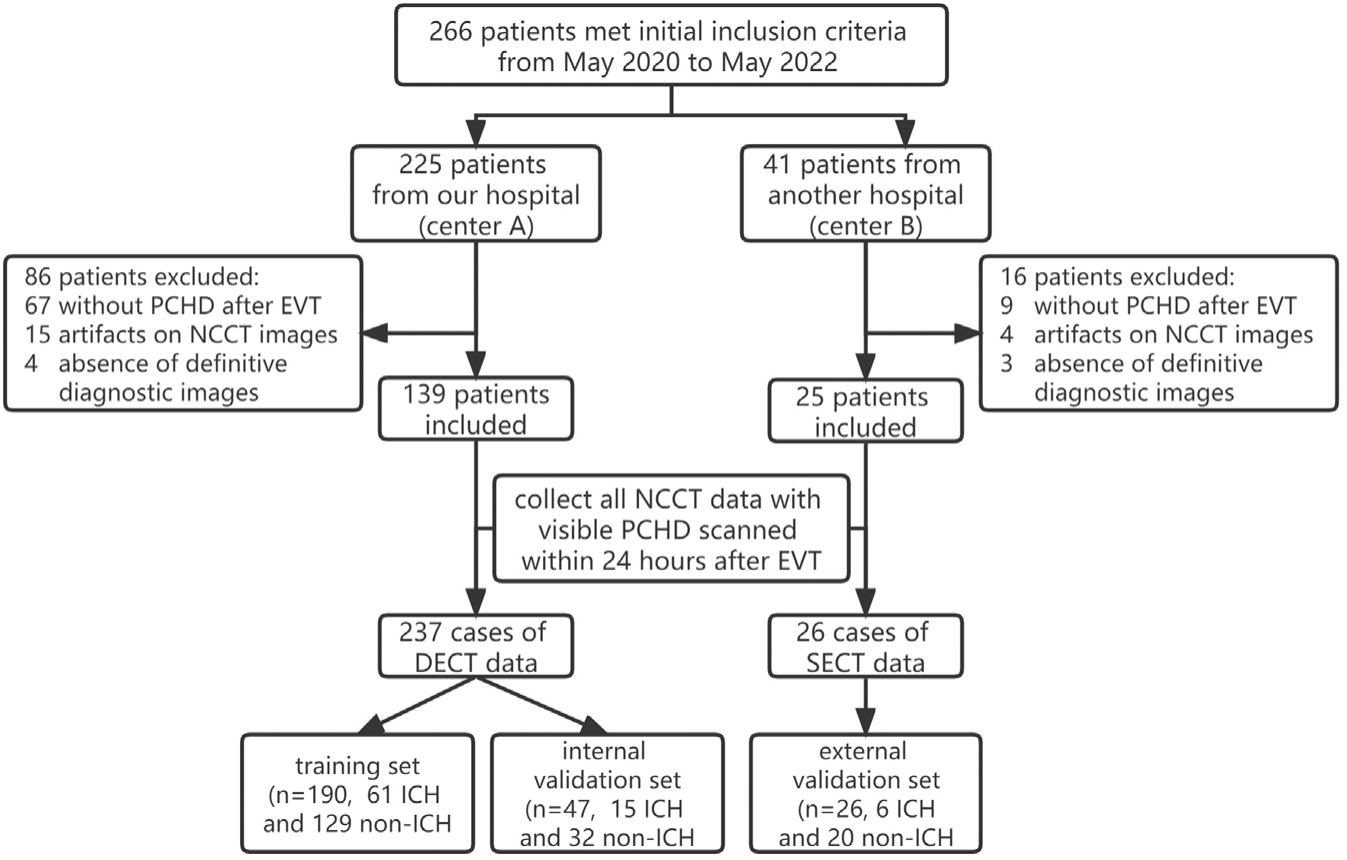
Flowchart of the study shows the recruitment pathway for patients and NCCT data selection for training and validation set. DECT, dual‐energy CT; EVT, endovascular thrombectomy; ICH, intracranial hemorrhage; NCCT, noncontrast CT; PCHD, postinterventional cerebral hyperdensity; SECT, single‐energy CT.

A total of 225 patients from Center A and 41 patients from Center B met the initial inclusion criteria. A total of 102 patients were excluded for the following reasons: no PCHD on post‐EVT NCCT images (*n* = 75, including 67 from Center A and 9 from Center B), artifacts affecting diagnosis (*n* = 19, including 15 from Center A and 4 from Center B), or absence of definitive diagnostic images (*n* = 7, including 4 from Center A and 3 from Center B). A total of 139 patients from Center A and 25 patients from Center B were ultimately included. The baseline characteristics of patients from the two centers are compared in Table 1. After collecting all NCCT images with visible PCHD from the included patients scanned within 24 h after EVT, 237 DECT data from Center A were randomly allocated to a training set (*n* = 190) and an internal validation set (*n* = 47). The 26 SECT data from Center B were used as an external validation set (*n* = 26). There was no statistically significant difference in the proportion of ICH in the included DECT images between Centers A and B (61/190 vs. 6/26, *p* > 0.05). The flowchart of this study is shown in Figure 2.

**TABLE 1:** Demographic and clinical data.

Characteristic	Dataset A	Dataset B	*p*
Sample size (*n*)	139	25	
Male, *n* (%)	79 (56.8%)	15 (60.0%)	0.77
Age (years)	68.8 ± 12.8	67.2 ± 15.3	0.58
Comorbid conditions			
Hypertension, *n* (%)	98 (70.6%)	15 (60.0%)	0.30
Diabetes mellitus, *n* (%)	26 (18.7%)	5 (20.0%)	0.88
Atrial fibrillation, *n* (%)	67 (48.2%)	8 (32.0%)	0.14
Prior stroke or TIA, *n* (%)	27 (19.4%)	5 (20.0%)	0.95
Smoking, *n* (%)	28 (20.1%)	5 (20.0%)	0.99
Clinical variables			
Baseline NIHSS score	18 (13–22)	21 (14.5–30.5)	0.14
Anterior circulation stroke, *n* (%)	120 (86.3%)	24 (96.0%)	0.18
Intravenous thrombolysis, *n* (%)	26 (18.7%)	4 (16.0%)	0.75
Oral anticoagulants, *n* (%)	17 (12.2%)	5 (20.0%)	0.30
Onset to puncture (min)	374.4 (288.0–547.2)	201.6 (266.4–716.4)	0.07
Puncture to reperfusion (min)	86.4 (43.2–144.0)	57.6 (43.2–165.6)	0.56

*Note:* Continuous variables were compared using the Mann–Whitney U test and Student's *t*‐test as appropriate and categorical variables were compared using Pearson's *χ* ^2^ test.

Abbreviations: NIHSS, National Institutes of Health Stroke Scale; TIA, transient ischemic attack.

**FIGURE 3:**
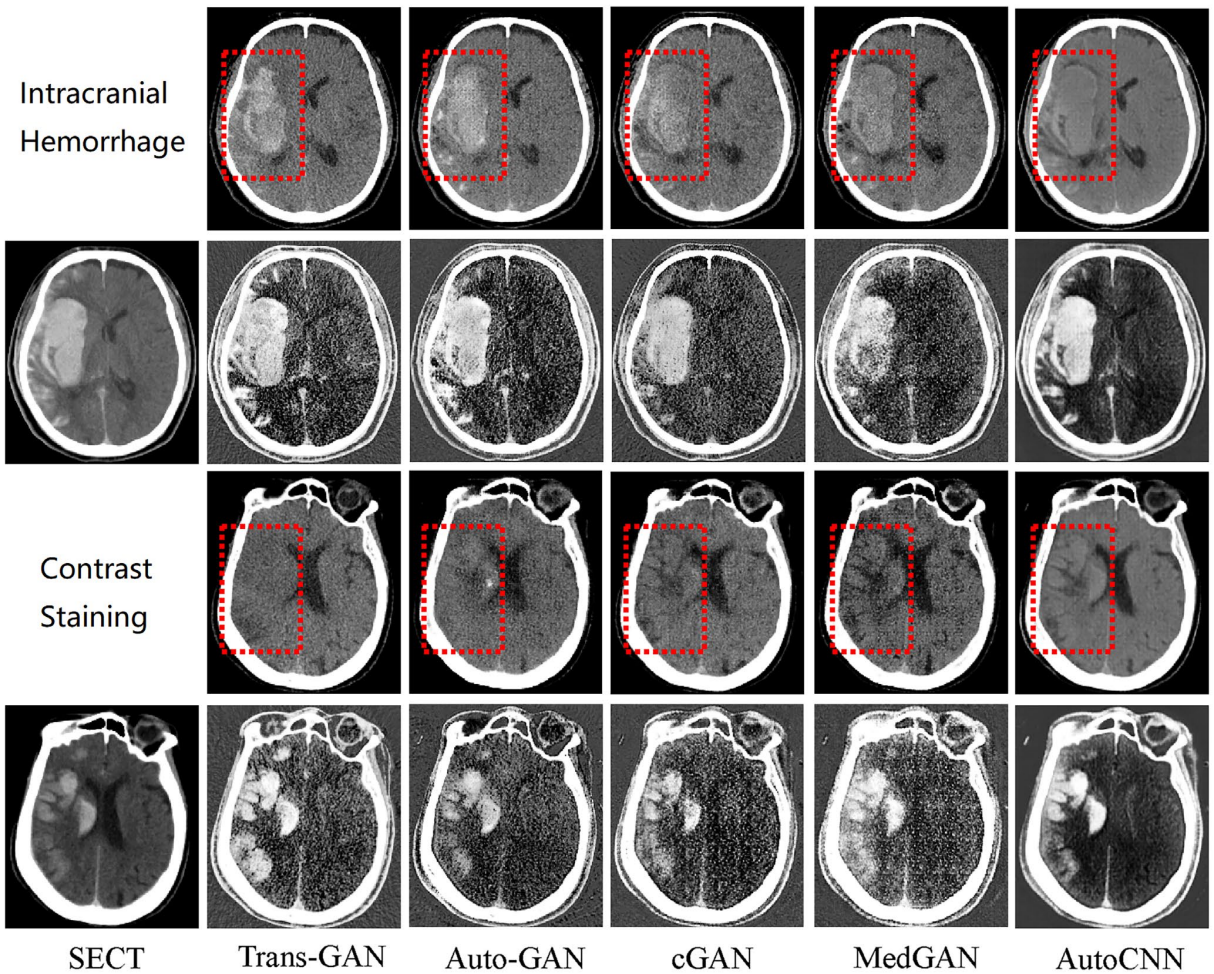
Visual comparison of SECT on two typical cases. In each case, the first row and second row show the VNC and IOM, respectively, and from left to right are the input simulated conventional CT images, and five images generated by our trans‐GAN and four other methods. Red boxes show areas for detailed comparison. IOM, iodine overlay map; trans‐GAN, transformer‐based generative adversarial network, VNC, virtual noncontrast.

The quantitative evaluation results for virtual material decomposition images generated by trans‐GAN, Autocontext CNN, cGAN, MedGAN, and AutoGAN are presented in Table 2. Overall, trans‐GAN demonstrated superior performance, achieving a PSNR of 28.54 ± 0.88 dB and an SSIM of 93.22% ± 0.93% for sVNC images, and a PSNR of 24.67 ± 0.97 dB and an SSIM of 87.65% ± 1.17% for sIOM images. These results represent the highest metrics among the five deep‐learning models evaluated. Furthermore, compared with the suboptimal AutoGAN, trans‐GAN showed a significant improvement in both PSNR and SSIM metrics. Moreover, we provided a visual comparison of two typical examples of ICH and CS in Figure 3, from which we can see the following: (1) the synthetic MDI generated by our trans‐GAN exhibited reduced noise and artifacts, with clearer structural details, particularly in high‐density regions such as hemorrhagic areas; (2) in the CS cases, only the sVNC images generated by trans‐GAN led to correct diagnosis, while others still retained high‐density areas (see red box in Figure 3) that led to wrong diagnosis (i.e., diagnosed as ICH). These results suggest that our trans‐GAN is superior to these state‐of‐the‐art methods.

**TABLE 2:** Quantitative comparison of trans‐GAN with several state‐of‐the‐art generation methods.

	sVNC		sIOM	
	PSNR (dB)	SSIM (%)	PSNR (dB)	SSIM (%)
Autocontext CNN	22.32 ± 1.98	85.46 ± 1.55	20.84 ± 1.58	81.26 ± 1.75
cGAN	22.97 ± 1.45	85.88 ± 1.36	21.31 ± 1.19	81.75 ± 1.62
MedGAN	24.13 ± 1.25	86.76 ± 1.15	22.12 ± 1.04	82.07 ± 1.43
AutoGAN	23.32 ± 1.12	87.15 ± 1.15	22.67 ± 0.98	83.34 ± 1.36
Trans‐GAN	28.54 ± 0.88	93.22 ± 0.93	24.67 ± 0.97	87.65 ± 1.17

Abbreviations: PSNR, peak signal‐to‐noise ratio; sIOM, synthetic iodine overlay map; SSIM, structural similarity; sVNC, synthetic virtual noncontrast; trans‐GAN, transformer‐based generative adversarial network.

**FIGURE 4:**
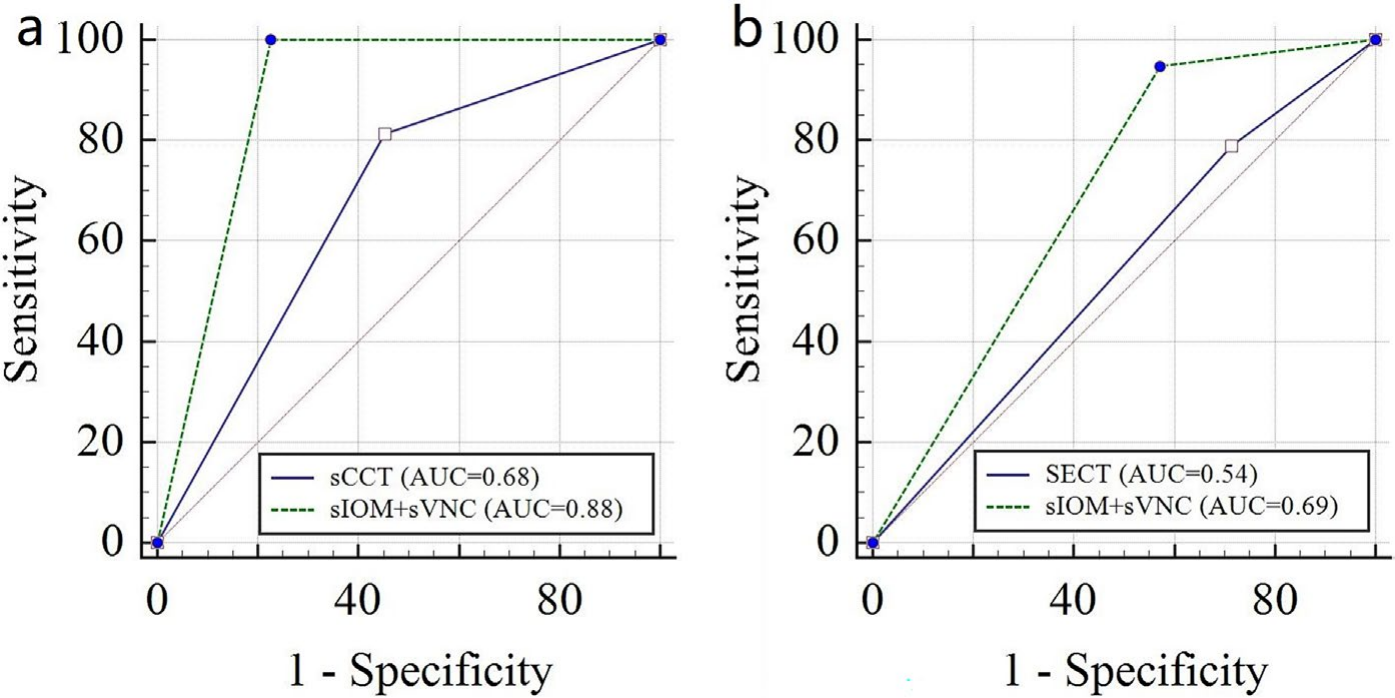
Receiver operating characteristic curves of the input images and the output images generated from the transformer‐based generative adversarial network in the internal validation set (a) and external validation set (b). sCCT, simulated conventional CT; SECT, single‐energy CT; sIOM, synthetic iodine overlay map; sVNC, synthetic virtual noncontrast.

In terms of the qualitative interpretation of images, the sensitivity, specificity, predictive value, and AUCs of input and output images from two validation sets are shown in Table 3. In the internal validation set, the AUC for the diagnosis of PCHD using sIOM and sVNC was 0.88 (95% CI 0.76–0.96), which was higher than the AUC of 0.68 (95% CI 0.53–0.81) for the input sCCT images. A DeLong test showed a significant difference between the two (*p* = 0.01). In the external validation set, the AUC for diagnosing PCHD using sIOM and sVNC was 0.69 (95% CI 0.48–0.85), surpassing the AUC of 0.54 (95% CI 0.33–0.73) for SECT images. While the DeLong test did not show statistical significance (*p* = 0.07), the trans‐GAN‐generated images demonstrated potential for providing improved diagnostic performance and supporting clinical decision‐making. The above receiver operating characteristic curves are shown in Figure 4. Additionally, in both the internal and external validation sets, the kappa values for PCHD diagnosis using the output images (*κ* = 0.83 [95% CI 0.67–0.98], *κ* = 0.56 [95% CI 0.19–0.94]) were higher than those using the input images (*κ* = 0.51 [95% CI 0.17–0.85], *κ* = 0.31 [95% CI 0.08–0.53]), demonstrating better inter‐reader agreement with synthetic MDI.

**TABLE 3:** Performance comparison of the PCHD diagnosis by using input and output images.

		Accuracy	Sensitivity	Specificity	Positive predictive value	Negative predictive value	AUC
Internal validation set	Input sCCT	0.64 (0.49–0.77)	0.81 (0.54–0.96)	0.55 (0.36–0.73)	0.48 (0.37–0.59)	0.85 (0.66–0.94)	0.68 (0.53–0.81)
	Output sVNC and sIOM	0.85 (0.72–0.94)	1.00 (0.79–1.00)	0.77 (0.59–0.90)	0.70 (0.54–0.81)	1.00 (0.79–1.00)	0.88 (0.76–0.96)
External validation set	Input SECT	0.65 (0.44–0.82)	0.79 (0.54–0.94)	0.29 (0.04–0.71)	0.75 (0.64–0.83)	0.33 (0.10–0.68)	0.54 (0.33–0.73)
	Output sVNC and sIOM	0.81 (0.61–0.93)	0.95 (0.74–0.99)	0.43 (0.10–0.82)	0.82 (0.70–0.90)	0.75 (0.28–0.96)	0.69 (0.49–0.85)

Abbreviations: AUC, area under the receiver to operating characteristic curve; PCHD, postinterventional cerebral hyperdensity; sCCT, simulated conventional CT; sIOM, synthetic iodine overlay map; sVNC, synthetic virtual noncontrast.

## 4 Discussion

In this study, we developed a transformer‐based generative adversarial network (trans‐GAN) to simultaneously generate sVNC and sIOM images from SECT or sCCT images. Compared with other advanced image generation networks, the images generated by this network closely resemble real images based on objective metrics. In addition, synthetic MDI generated by the network showed higher AUC and better inter‐reader consistency for real‐time diagnosis of PCHD compared with the input SECT.

PCHD is not uncommon after EVT, and its nature is directly linked to planning life‐saving treatments such as anticoagulation or antiplatelet therapy [7, 28]. Therefore, identifying the nature of PCHD in the early post‐EVT period to differentiate between ICH and CS is crucial. However, since ICH and CS have similar attenuation coefficients, distinguishing between the two within the first 24 h of EVT using NCCT with a single‐energy spectrum is challenging [29]. Therefore, some studies have attempted to identify SECT imaging markers that can differentiate the nature of PCHD, such as absolute and relative iodine concentration and metallic hyperdensity signs [30, 31]. However, due to the unknowable property of real‐time diagnosis of PCHD on SECT, these studies have used follow‐up results beyond 24 h after EVT as the diagnostic gold standard. Therefore, the results obtained from these studies are more predictive in nature rather than reflecting real‐time diagnosis. In image interpretation, although the external validation AUC of sIOM and sVNC in diagnosing PCHD was higher than that of the input images, the DeLong test did not show a statistically significant difference, which may be one of the reasons. Currently, the real‐time diagnostic efficacy of VNC and IOM images based on the DECT material separation principle for PCHD is widely recognized, and sCCT images reconstructed from DECT images can be used as equivalents to real SECT images [32, 33]. The innovation of this study lies in the use of multiple sets of matched IOM, VNC, and sCCT images for network training and the first attempt to directly generate IOM and VNC images from SECT images that can be used for real‐time differentiation between ICH and CS, thereby advancing the diagnosis time window for PCHD from the usual 2 days or more after EVT to immediately after NCCT examination within 24 h post‐EVT.

With the continuous advancement of deep‐learning technology, some studies have attempted to apply this approach to optimize the diagnosis process for HT in AIS patients after EVT [34]. One of these studies has already constructed a model based on preoperative MR images to predict post‐EVT HT [35]. However, that study also used the final diagnosis obtained from a follow‐up examination 2–7 days after EVT as the outcome indicator for model training, making the model unable to make a real‐time differential diagnosis of ICH or CS for PCHD within 24 h after EVT. Additionally, some studies have suggested that post‐EVT imaging biomarkers may be more suitable for predicting HT compared with preoperative images [36]. In this study, we chose to generate synthetic MDI based on post‐EVT SECT instead of directly predicting the diagnostic results [37, 38]. That is because, on the one hand, compared with generating a binary diagnostic result of whether HT is present or absent, the sVNC and sIOM images generated by the network can further provide important information, such as the extent and grading of HT. On the other hand, this approach allows the diagnostic authority for PCHD to be given to doctors rather than the network, which is closer to the actual DECT‐based diagnostic process in clinical practice and, therefore, yields results that are more subjectively convincing.

The present study has several limitations. First, although it is a two‐center study, the sample size collected retrospectively is still relatively small, which may lead to selection bias. Further studies with larger sample sizes are needed. Secondly, due to the inability to directly diagnose PCHD in real‐time using SECT images, the diagnostic gold standard for image interpretation differed between the internal and external validation sets in this study. For ethical considerations, we were unable to directly compare DECT‐derived VNC and iodine maps with SECT trans‐GAN‐generated synthetic MDI in the same patient at the same time point. In the future, we plan to address this limitation through animal experiments or alternative study designs that allow such comparisons under controlled conditions. Third, the network constructed in this study currently only generates synthetic MDI for input NCCT images containing PCHD within 24 h after EVT. Therefore, the application value of this study's network should be extrapolated with caution.

In conclusion, our study developed an advanced trans‐GAN using deep learning to generate synthetic MDI from input SECT images for real‐time discrimination and diagnosis of ICH and CS within 24 h after EVT. The output images generated by the network demonstrate higher AUC and inter‐reader consistency for diagnosing PCHD than the input SECT images. This study provides a new approach for the real‐time detection of ICH using SECT in hospitals without DECT capabilities.

## Author Contributions

Concept and design: Z.D., D.S., and T.W; Acquisition, analysis, or interpretation of data: T.W., C.J., W.D., and Q.C; Drafting of the manuscript, statistical analysis: T.W. and C.J; Critical revision of the manuscript for important intellectual content, supervision: Z.D. and D.S. Obtained funding: Z.D. T.W. and C.J. contributed equally to the manuscript. D.S. and Z.D. are both senior authors.

## Ethics Statement

This study was approved by the Ethics Committee of the Affiliated Hangzhou First Hospital, Westlake University School of Medicine (No: IIT‐20221212‐0198‐01). Patient consent was waived due to the retrospective nature of the study.

## Conflicts of Interest

D.S. is an employee of Shanghai United Imaging Intelligence Co. Ltd. The companies have no role in designing and performing the surveillance and analyzing and interpreting the data. All other authors report no conflicts of interest relevant to this article.

## Supporting information


Appendix S1.

## Data Availability

The data that support the findings of this study are available on request from the corresponding author. The data are not publicly available due to privacy or ethical restrictions.
