# Supplementary material for: Deep‐Learning Generated Synthetic Material Decomposition Images Based on Single‐Energy CT to Differentiate Intracranial Hemorrhage and Contrast Staining Within 24 Hours After Endovascular Thrombectomy
Source: CNS Neurosci Ther. 2025 Jan 24;31(1):e70235. doi: 10.1111/cns.70235 (PMC11758448; doi:10.1111/cns.70235)
Supplement: Supplementary file 1 — Appendix S1. [file CNS-31-e70235-s001.docx]

**SUPPLEMENTAL MATERIAL**

**Deep-Learning Generated Synthetic Material Decomposition Images Based on Single-energy CT to Differentiate Intracranial Hemorrhage and Contrast Staining Within 24 Hours After Endovascular Thrombectomy**

**Image acquisition**

At Medical Center A, DECT was performed 24 h after EVT using a dual-source 128-slice CT scanner (SOMATOM Flash, Siemens Healthcare, Forchheim, Germany). The scanning parameters were as follows: 2 bulb voltages: 80 kV and Sn140 kV; a reference current: 392/196 mAs; collimator width: 64 mm × 0.6 mm; automatic reconstruction layer thickness: 0.75 mm; layer spacing: 0.7 mm; scanning pitch: 0.7; rotation time: 0.5 s/r; fusion coefficient: 0.4. The raw spiral projection data were transferred to the post-processing workstation (Syngo. CT Dual-Energy Brain Hemorrhage; Siemens) to generate a simulated conventional CT (sCCT, equivalent to a 120-kV conventional single-energy CT) image, virtual non-contrast (VNC), and iodine overlay maps (IOM).

At Medical Center B, SECT was performed 24 h after EVT using a dual-source 64-slice CT scanner (SOMATOM Flash, Siemens Healthcare, Forchheim, Germany). The scanning parameters were as follows: bulb voltages: 120 kV; tube current: 340mAs; collimator width: 192 mm×0.6 mm; automatic reconstruction layer thickness: 5 mm; layer spacing: 5 mm; scanning pitch: 1.2, rotation time: 0.5 s/r.

**Definition of PSNR and SSIM**

Two metrics were used to assess the image quality of the generated VNC and ION, including peak signal-to-noise ratio (PSNR) and structural similarity (SSIM). Among them, PSNR is a ratio representing the maximum possible power of a signal to the power of destructive noise that affects the accuracy of its representation. Here, the signal refers to the original image, i.e., the ground truth, and the noise refers to the mean square error (MSE) of the synthesized image and the ground truth. The PSNR is calculated by using the following formula:

$$PSNR=20\cdot\log_{10} \left( \frac{{MAX\left( y \right)}}{\sqrt{MSE\left( y,\hat{y} \right)}} \right),$$

$$MSE\left( y,\hat{y} \right)=\frac{\sum_{i} {(y_{i}-\hat{y}_{i})}^{2}}{h\times w\times z} ,$$

where $y$ represents the ground truth and $\hat{y}$ represents the generated image. MAX is the maximum value of the ground truth. $h$, $w$, and $z$ represent the size of the ground truth, respectively.

SSIM is an image quality measure that represents the similarity between two images, which measures the similarity of images from three key features, including luminance, contrast and structure. Thus, this metric can better match human subjectivity. The SSIM is calculated by using the following formula:

$$SSIM=\frac{\left( 2\mu_{y}\mu_{y}+C_{1} \right)\left( 2\sigma_{yy}+C_{2} \right)}{\left( \mu_{y}^{2}+\mu_{y}^{2}+C_{1} \right)\left( \sigma_{y}^{2}+\sigma_{y}^{2}+C_{2} \right)} ,$$

$$C_{1}={(k_{1}L)}^{2},$$

$$C_{2}={(k_{2}L)}^{2},$$

where $\mu_{y}$ and $\mu_{y}$ represent the means of $y$ and $\hat{y}$, respectively. In addition, $\sigma_{y}$ and $\sigma_{y}$ represent the variances of $y$ and $y$, respectively, and $\sigma_{yy}$ represents their covariance. $L$ denotes the dynamic range of image pixel values; $k_{1}$ and $k_{2}$ are generally set to 0.01 and 0.03, respectively.

**Comparison Methods**

We compared our trans-GAN with four state-of-the-art generation methods, including auto-context CNN, cGAN, MedGAN, and Auto-GAN. The implementation details of each comparison method are as follows:

1. The auto-context CNN consists of four convolution layers.
2. The 3D-cGAN consists of a U-Net-like generator and a four-layer discriminator.

3) The MedGAN consists of a CasNet generator, a discriminator and a pre-trained feature extractor. The generator translates input images to the target domain through progressive refinement via encoder-decoder blocks. The discriminator is used to distinguish between real and transformed images and co-serve as a trainable feature extractor to calculate the modified perceptual loss. Finally, the pre-trained feature extractor extracts deep features to calculate style transfer losses.

4) The Auto-GAN consists of a translation network (to translate images from source to target domain), a self-representation network (to guide the decoder), and a discriminator. The self-representation network is an auto-encoder trained only by target images (VNC or IOM images). Once well-trained, the feature maps extracted from the decoder of the self-representation network are used to guide the optimization of the decoder of the translation network.

Note that only for TarGAN we can use one model to predict the VNC and IOM images from the SECT images at the same time; for the other methods, we need to use two identical models to predict the VNC and IOM images from the SECT images, respectively.
